# Supplementary material for: Splice-Junction-Based Mapping of Alternative Isoforms in the Human Proteome
Source: Cell Rep. Author manuscript; Available in PMC 2020 Jan 15. (PMC6961840; doi:10.1016/j.celrep.2019.11.026)
Supplement: 2 [file NIHMS1546469-supplement-2.zip › DF1/PXD010154/Thyroid/Thyroid_39_THBS1_VVNSTTGPGEHLRNALWHTGNTPGQVMMSLMLWTSVAPSSSTPK.pdf]

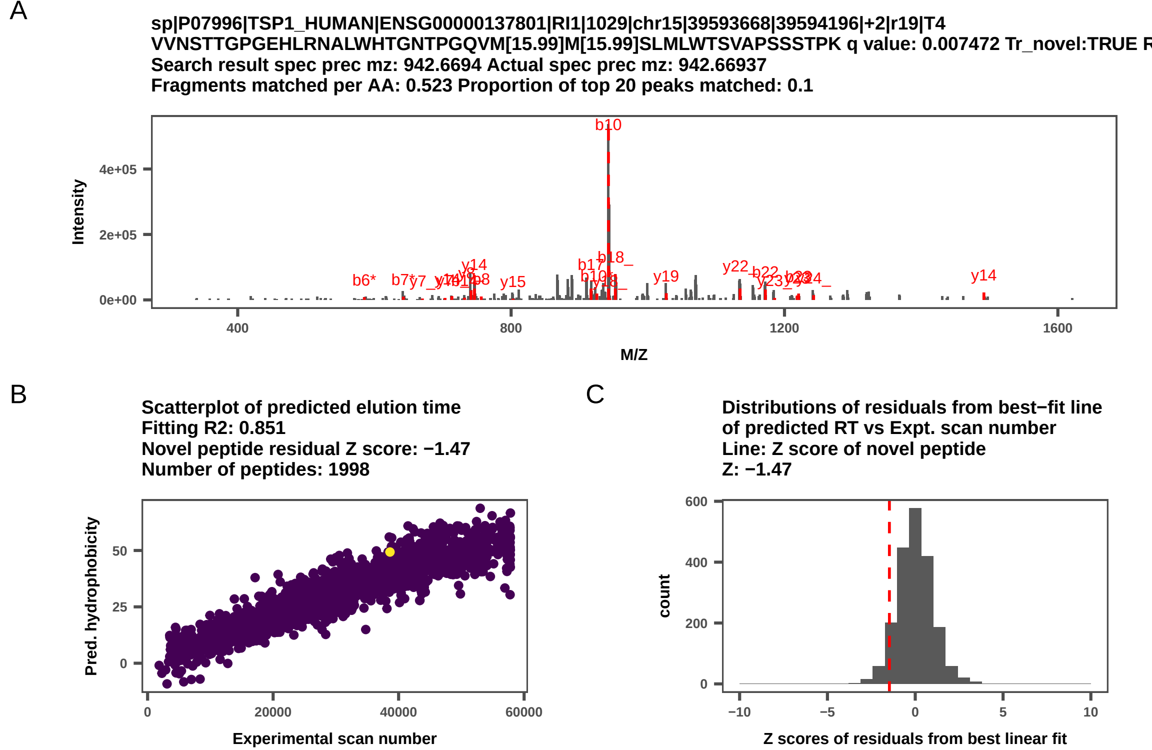
A
sp|P07996|TSP1_HUMAN|ENSG00000137801|RI1|1029|chr15|39593668|39594196|+2|r19|T4
VVNSTTGPGEHLRNALWHTGNTPGQVM[15.99]M[15.99]SLMLWTSVAPSSSTPK q value: 0.007472 Tr_novel:TRUE R
Search result spec prec mz: 942.6694 Actual spec prec mz: 942.66937
Fragments matched per AA: 0.523 Proportion of top 20 peaks matched: 0.1
Intensity
4e+05
2e+05
0e+00
b10
b6*
b7*
y7_
y14
y15
b17
b18_
y19
y22_
b22
y14
400
800
1200
1600
M/Z
B
Scatterplot of predicted elution time
Fitting R2: 0.851
Novel peptide residual Z score: −1.47
Number of peptides: 1998
Pred. hydrophobicity
50
25
0
0
20000
40000
60000
Experimental scan number
C
Distributions of residuals from best−fit line
of predicted RT vs Expt. scan number
Line: Z score of novel peptide
Z: −1.47
count
600
400
200
0
−10
−5
0
5
10
Z scores of residuals from best linear fit
